# Meta-analysis of clinical efficacy and safety of immunotherapy combined with chemotherapy in non-small cell lung cancer

**DOI:** 10.1515/med-2024-1071

**Published:** 2025-08-07

**Authors:** Wugang Zhu, Wenjing Xu, Degan Liu, Lei Wan, Xiaolan Lu

**Affiliations:** Department of Oncology, The Affiliated Xinghua People’s Hospital, Medical School of Yangzhou University, Xinghua, China; Department of Critical Care Medicine, The Affiliated Xinghua People’s Hospital, Medical School of Yangzhou University, 419 South Yingwu Road, Xinghua, 225700, China

**Keywords:** immunotherapy, combination chemotherapy, non-small cell lung cancer, meta-analysis

## Abstract

**Purpose:**

This study aimed to assess the clinical effectiveness and safety of combining immunotherapy with chemotherapy for non-small cell lung cancer.

**Methods:**

A comprehensive search of studies published until January 2024 was conducted. Quality assessment was performed using the NOS scale, and a meta-analysis was carried out with RevMan 5.4.1 software. Heterogeneity was assessed using the *Q*-test, and combined effects were calculated with fixed or random effects models. Results were visualized using forest plots, and a sensitivity analysis was performed.

**Results:**

Out of 1,061 sources, 11 met the inclusion criteria. The meta-analysis indicated that the combination treatment significantly improved 1-year overall survival, objective response rate, and disease control rate compared to chemotherapy alone (*P* < 0.05), with no significant difference in adverse reactions (*P* > 0.05). Immune function markers CD4+ and CD4+/CD8+ were higher, and CD8+ was lower in the combined treatment group. Sensitivity analysis confirmed the stability and reliability of the results (OR (95% CI) 3.72 (2.34–5.90), *P* < 0.00001), although publication bias was indicated by funnel plots.

**Conclusion:**

The combination of chemotherapy and immunotherapy exhibits the potential to enhance both survival rates and clinical effectiveness, without the concomitant rise in severe adverse reactions.

## 1 Introduction

Recently, a large number of clinical trials have verified the efficacy and safety of immunotherapy in malignant tumors, such as tumor-infiltrating lymphocytes, transgenic T lymphocytes expressing antigen-specific T cell receptors or chimeric antigen receptors, natural killer cells, cytokine-induced killer cells, and dendritic cells, providing new therapeutic strategies for hematological and solid tumors. Based on the data published by the World Health Organization’s International Agency for Research on Cancer in 2023 [1], although lung cancer incidence has decreased globally, it remains the leading cause of cancer-related deaths, comprising approximately 18% of the total number of fatalities [2]. Notably, China recorded approximately 787,000 new cases of lung cancer in 2015, which is equivalent to over 2,100 new cases per day [3]. Among the various types of lung cancer, non-small cell lung cancer (NSCLC) dominates, representing approximately 85% of all cases [2].

Despite the availability of various treatment options for lung cancer, such as surgical resection, chemotherapy, radiotherapy, and targeted therapy, their overall clinical efficacy remains unsatisfactory, leading to a poor prognosis. Five-year survival rates after treatment are less than 15% [2]. Furthermore, many tumors are diagnosed at advanced stages, missing the optimal window for surgical intervention and leaving patients with limited options, such as chemotherapy. Unfortunately, these treatments often come with significant adverse reactions that cause irreversible harm to patients [4,5]. In recent years, the emergence of tumor immunotherapy, specifically through the use of immune checkpoint inhibitors like programmed cell death protein-1 or programmed cell death-ligand 1, and cytotoxic T lymphocyte-associated antigen 4, has shown promising results. These inhibitors effectively modulate excessive immune responses induced by tumors, thereby promoting long-term anti-tumor effects, leading to improved disease response rates and outcomes for patients [6–11]. Although some studies have indicated the superiority of combining immunotherapy with chemotherapy over chemotherapy alone for NSCLC patients [12–16], these studies often suffer from limitations such as small sample sizes, retrospective design, and lack of large-scale clinical trials. Therefore, the application of meta-analysis methods becomes crucial in quantitatively assessing the efficacy and safety of combining immunotherapy with chemotherapy for NSCLC treatment, providing a solid evidence-based foundation for clinical decision-making.

## 2 Materials and methods

### 2.1 Sources of materials and retrieval strategies

In order to gather relevant information, a comprehensive computerized search was performed using several databases including China Knowledge Net, Wanfang, Weipu Chinese scientific journals, Chinese Biomedicine, PubMed, Web of science, Cochrane library, etc. The search period covered the database establishment date up until November 2023. The search strategy in Chinese included the keywords “immunotherapy,” “chemotherapy,” “non-small cell lung cancer,” and “curative effect,” while synonyms were also considered. Similarly, the English search strategy involved the terms “immunotherapy,” “chemotherapy,” “non-small cell lung cancer,” and “curative effect.”

### 2.2 Inclusion and exclusion criteria

Criteria for literature inclusion: (1) patients included in the study were diagnosed with NSCLC; (2) the experimental group received a combination of immunotherapy and chemotherapy, while the control group received chemotherapy alone. There were no restrictions on the specific types and treatment regimens of immunotherapy and chemotherapy; (3) the outcome measures assessed were overall survival (OS), objective response rate (ORR), disease control rate (DCR), adverse reactions, CD4+, CD8+, and CD4+/CD8+; and (4) in case of multiple publications by the same author reporting the same data, the study with the largest sample size or the most recent publication were chosen.

The following criteria were applied to the exclusion of literature: (1) removal of duplicate and irrelevant studies as well as review literature; (2) exclusion of non-randomized controlled trials; (3) elimination of studies with inconsistent outcome indicators; (4) exclusion of trials where the experimental group does not receive immunotherapy combined with chemotherapy or the control group receives chemotherapy alone; (5) omission of studies with missing, incomplete, unavailable, or obviously incorrect data; and (6) exclusion of literature studies conducted before 2020 with a sample size smaller than 70.

### 2.3 Literature screening and data extraction

Two researchers from the research team conducted a thorough review of the literature using predefined inclusion and exclusion criteria. Initially, they assessed the titles and abstracts of the articles and, if necessary, proceeded to examine the full text. In cases where there were discrepancies, they consulted external experts to reach a consensus. Articles that met the inclusion criteria were carefully analyzed using a pre-established literature characteristics table to extract pertinent information. This included details such as study design, total sample size, sample size of the experimental group, sample size of the control group, and outcome measures, among others.

### 2.4 Literature quality evaluation

In order to assess the methodological quality, we utilized the Newcastle-Ottawa quality assessment scale (NOS). The scoring system ranged from 0 to 9, where scores falling within the range of 7–9 were indicative of high methodological quality (grade A), scores ranging from 4 to 6 reflected fair methodological quality (grade B), and scores below 4 indicated low methodological quality (grade C).

### 2.5 Statistical methods

Literature management was conducted using Note Express 3.2 software, while Excel2003 software was utilized for literature data collection and extraction. Revman 5.4.1 software was employed for performing the meta-analysis. Heterogeneity analysis was conducted on the extracted data using the *Q*-test (*P*-value), with the addition of the *I* ^2^ value to assess heterogeneity. In the case where *P* > 0.10 or *I* ^2^ ≤ 50%, indicating the absence of heterogeneity, a fixed-effect model (FEM) was applied. In contrast, if heterogeneity was present, a random-effects model (REM) was used. At the same time, the source of literature heterogeneity was explored by deleting the literature with the largest proportion of weight. The pooled results of the meta-analysis were described using the odds ratio (OR) and its corresponding 95% confidence interval (CI), while illustrating forest plots. Sensitivity analysis was conducted to assess the stability of the meta-analysis results, and publication bias was evaluated through the use of funnel plots. The significance level for testing was set at *α* = 0.05 (two-sided).

## 3 Results

### 3.1 Literature search results

**Figure 1:**
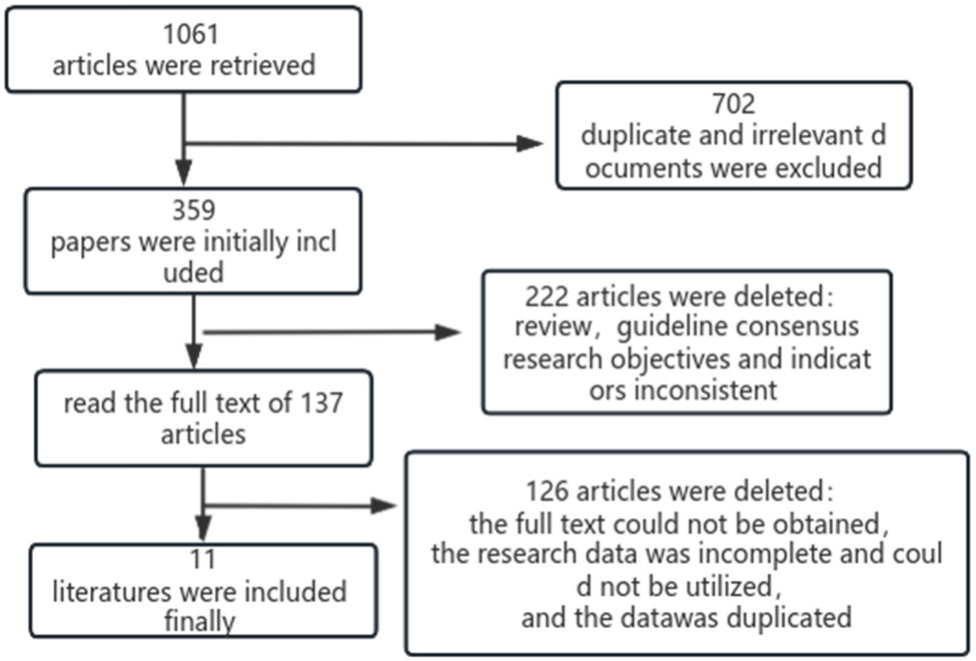
Flow chart of literature screening.

Based on the article search strategy, a total of 1,061 pertinent documents were initially retrieved from various databases such as China Knowledge Net, Wanfang Database, VIP Chinese Science and Technology Journal Database, China Biomedical Database, PubMed, Web of Science, Cochrane library, among others. To eliminate duplicate literature, duplicates found in multiple databases were removed. Subsequently, through careful examination of title, abstract, and full text, 11 pertinent literatures were ultimately included in the study. The literature screening process is illustrated in Figure 1.

### 3.2 Basic characteristics and quality evaluation of literature

The baseline data comprise primarily information such as gender, age, disease duration, treatment plan, and outcome measures. A comprehensive overview of the baseline data can be found in the 11 literature sources included in this study. The quality of the 11 selected studies is assessed using the NOS scale, with the results presented in Table 1 [17–27].

**Table 1:** Basic characteristics and quality evaluation table of literature

First author	Year of publication	Sample size (example)		Age	Intervention measures		Outcome index	NOS
		Test group	Control group		Control group	Test group		
He Jing [17]	2022	150	130	40 years old and 80 years old	PD-1/PD-L1 immunization combined with docetaxel chemotherapy	Docetaxel chemotherapy	①→④⑤⑥⑦⑧	5
Yang Juyin [18]	2023	40	40	/	Single chemotherapy group combined with PD-1/PD-L1 inhibitor	AP regimen containing platinum (pemetrexed + platinum) or TP regimen (paclitaxel + platinum)	②→③	5
Zhao Jingjing [19]	2022	44	44	24 years old and 71 years old	DC/CIK immunotherapy was performed on the basis of simple chemotherapy	Simple chemotherapy	③→③⑥⑦⑧	5
Sun Liling [20]	2023	38	44	18 years old, 75 years old	Carrell monoclonal antibody combined with platinum-containing double drugs	Platinum-containing dual drug therapy	①→②③④⑥⑦⑧	6
Li et al. [21]	2023	50	50	60–83 years old	Carrilizumab combined with chemotherapy	Chemotherapy	④→	5
Chen Min [22]	2023	52	52	Test group: 60.47 ± 5.13; control group: 61.58 ± 5.44	Chemotherapy group was immunized with pablizumab on the basis of chemotherapy	Give	②→③④⑥⑧	6
Sun Xiongying [23]	2022	36	35	The experimental group: 68.13 ± 4.28; the control group: 68.35 ±	Immunotherapy combined with chemotherapy	Platinum-containing dual-drug chemotherapy	⑤→⑧	5
He Siyi [24]	2021	35	35	4.32	Albumin paclitaxel + xindiril monoclonal antibody	Simple chemotherapy	③→③⑥⑦⑧	5
Pan Enyuan [25]	2021	40	40	Experimental group: 57.12 ±	On the basis of chemotherapy, combined with bevacizumab	Albumin paclitaxel chemotherapy regimen	⑥→⑧	5
Shiyuan Garden [26]	2023	38	35	7.05; control group: 56.41 ± 7.14	Chemotherapy plus immunotherapy	Pemetrexed plus platinum chemotherapy	④→	5
Zhang Jingbao [27]	2022	75	75	/	A single bead for Riley	Simple chemotherapy	②③⑤⑥⑦⑧	5

Notes: ① 1 years of OS, ② ORR, ③ DCR, ④ PFS, ⑤ adverse reactions, ⑥ CD4+, ⑦ CD8+, ⑧ CD4+/CD8+.

### 3.3 Meta-analysis results

#### 3.3.1 Clinical efficacy

##### 3.3.1.1 One-year OS

**Figure 2:**
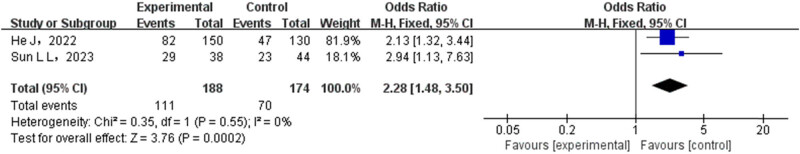
Forest map of original 1-year OS comparison between test group and control group.

Two literature reviews were conducted to compare the OS rates of patients in the experimental and control groups over a period of 1 year. The test group consisted of 188 cases, while the control group had 174 cases. Statistical heterogeneity tests were performed on the included literature, and the results indicated no significant variation across different studies. This allowed for the utilization of FEM to combine the data from the literature reviews. The meta-analysis showed significantly higher OS rates in the experimental group (OR = 2.28, 95% CI (1.48–3.50, 0.0002)), as depicted in Figure 2.

##### 3.3.1.2 ORR

**Figure 3:**
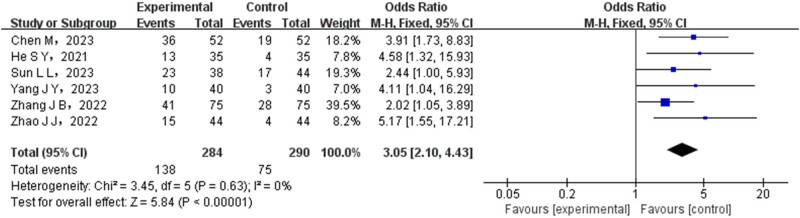
Forest map of ORR comparison between test group and control group.

In total, six articles conducted a comparison between the ORR of patients in the experimental group and the control group. The experimental group consisted of 284 cases, whereas the control group had 290 cases. A heterogeneity test was performed on the collected literature, and the results indicated that there was no statistical heterogeneity among the different studies. Therefore, a FEM was utilized to combine the data from these studies. The meta-analysis showed significantly higher ORR rates in the experimental group, with a statistically significant difference (OR = 3.05, 95% CI (2.10–4.43), *P* < 0.00001), as illustrated in Figure 3.

##### 3.3.1.3 DCR

**Figure 4:**
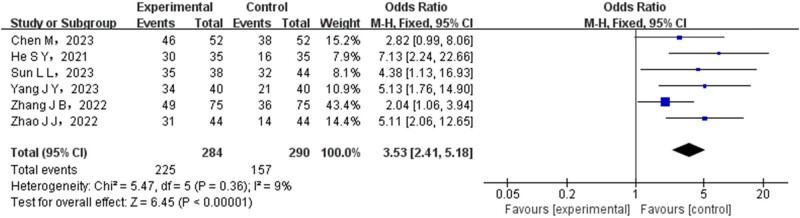
Forest map of DCR comparison between test group and control group.

A total of six studies were included in the analysis to compare the DCR of patients in the experimental group and the control group. The experimental group consisted of 284 cases, while the control group had 290 cases. Statistical tests were conducted to assess the heterogeneity of the included studies, and the results indicated no significant heterogeneity among them. Therefore, FEM was utilized to combine the data from these studies. The meta-analysis showed significantly higher DCR rates in the experimental group (OR = 3.53, 95% CI (2.41–5.18), *P* < 0.00001), as depicted in Figure 4.

##### 3.3.1.4 Progression-free survival (PFS)

**Figure 5:**
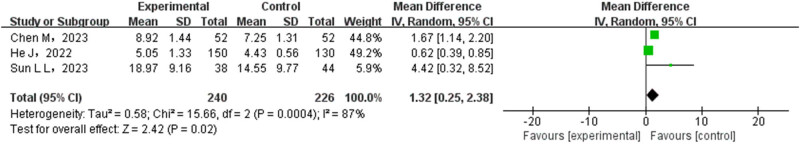
Forest map of PFS comparison between test group and control group.

The PFS of patients in the experimental group was compared to that in the control group in a total of three literatures. A total of 240 cases were included in the test group and 226 cases in the control group. The included literature underwent a heterogeneity test, which yielded a result of 0.0004, indicating statistical heterogeneity among the different studies. Therefore, a REM was utilized to combine the literature data. The meta-analysis showed significantly longer PFS rates in the experimental group (OR = 1.32, 95% CI (0.25–2.38), PP 0.02), as depicted in Figure 5.

##### 3.3.1.5 Adverse reaction

**Figure 6:**
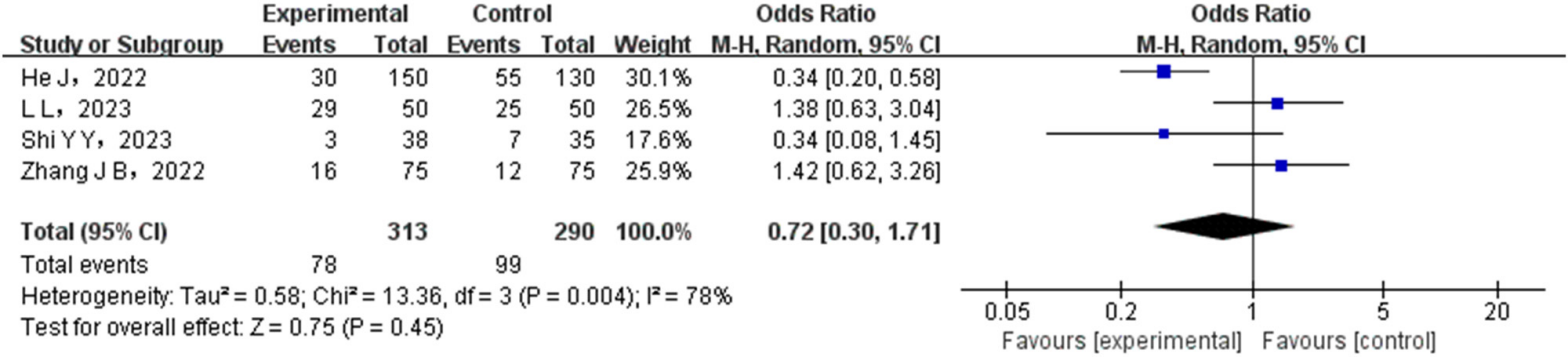
Forest map of adverse reactions between the test group and the control group.

There were a total of four studies that conducted a comparison of adverse reactions between the experimental and control groups. The test group consisted of 313 cases, while the control group had 290 cases. Statistical heterogeneity testing was performed on the included studies, yielding a result of 0.004%, indicating the presence of significant heterogeneity among them. Therefore, a REM was employed to combine the data from these studies. The meta-analysis results revealed no statistically significant difference in adverse reactions between the test group and control group (*P* > 0.05), as illustrated in Figure 6.

#### 3.3.2 Immunity

##### 3.3.2.1 CD4+

**Figure 7:**
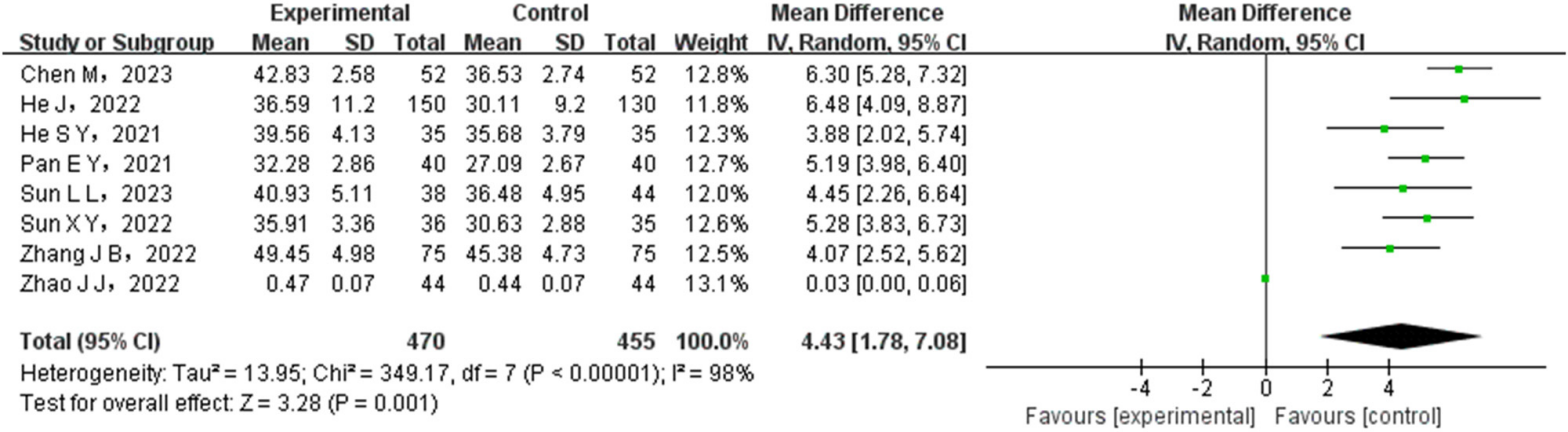
Forest map of CD4+ comparison between test group and control group.

A total of eight studies were conducted to compare the CD4+ levels between the experimental and control groups. The test group consisted of 470 cases, while the control group had 455 cases. A heterogeneity test was conducted on the included studies, revealing statistical heterogeneity among them. Hence, a REM was employed to combine the data from these studies. The meta-analysis showed significantly higher CD4+ rates in the experimental group (OR = 4.43, 95% CI (1.78–7.08), PSA 0.001), as illustrated in Figure 7.

##### 3.3.2.2 CD8+

**Figure 8:**
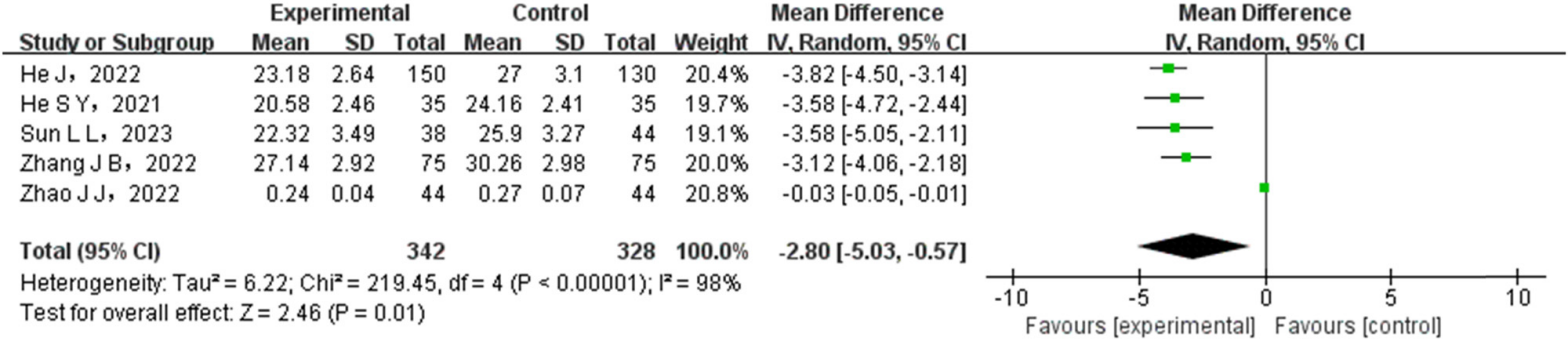
Forest map of CD8+ comparison between test group and control group.

A total of five articles conducted a comparison between the experimental group and the control group regarding the CD8+ level. Among these studies, the number of cases in the experimental group was 342, while in the control group it was 328. The pooled data were subjected to a heterogeneity test, which revealed that there was a significant variability among the different literature studies. To address this, the REM was employed for data synthesis. The meta-analysis showed significantly lower CD8+ rates in the experimental group (OR = −2.80, 95% CI (−5.03 to 0.57), PP01), as illustrated in Figure 8.

##### 3.3.2.3 CD4+/CD8+

**Figure 9:**
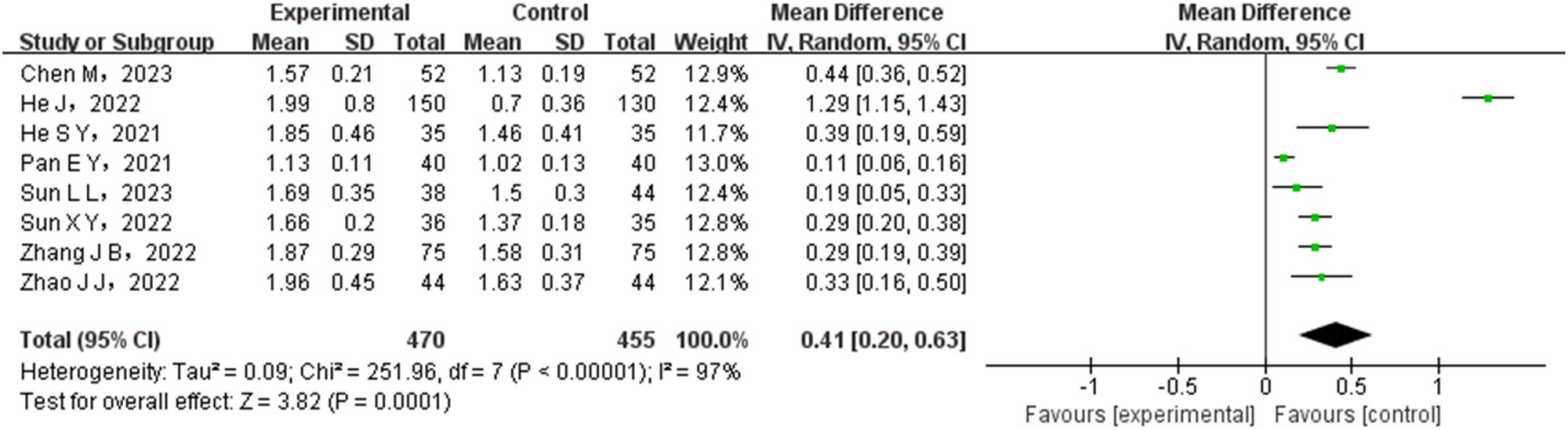
Forest map of CD4+/CD8+ comparison between test group and control group.

A total of eight research papers conducted a comparison between the experimental group and the control group in terms of the CD4+/CD8+ level. Among them, the test group consisted of 470 cases, while the control group consisted of 455 cases. Statistical heterogeneity was detected among the included papers, indicating variations in the study results. Therefore, a REM was utilized to combine the data from different studies. The meta-analysis showed significantly higher CD4+/CD8+ level rates in the experimental group (OR = 0.41, 95% CI (0.20–0.63), *P* < 0.0001). This finding is depicted in Figure 9.

### 3.4 Sensitivity analysis and literature bias test

**Figure 10:**
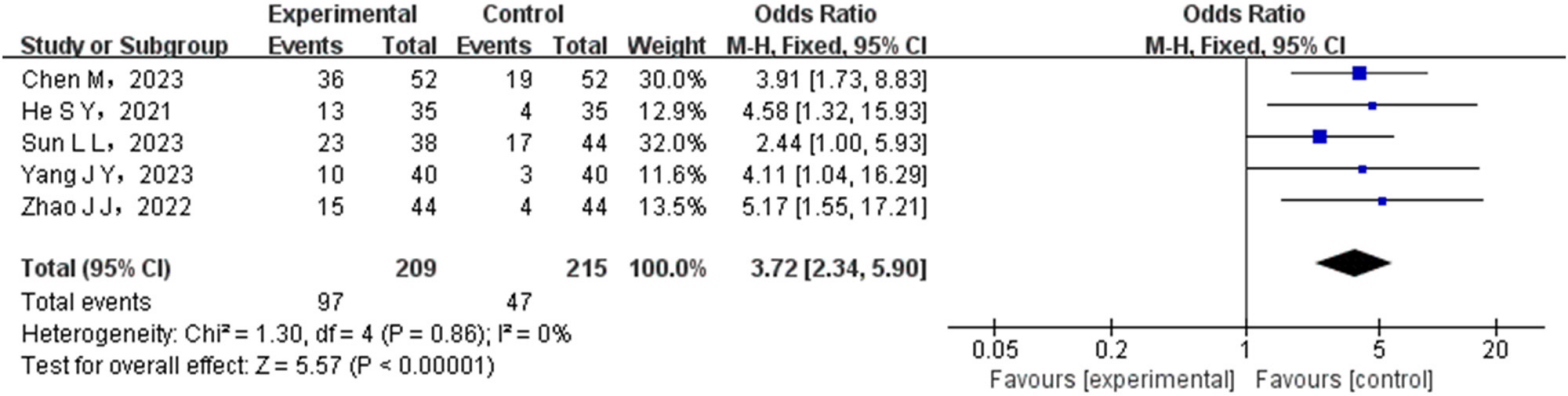
Forest map of sensitivity analysis between test group and control group.

Sensitivity analysis was conducted using ORR as a case study to validate the reliability of the findings. By excluding the literature with the highest proportion and integrating the literature effect again, the OR (95% CI) was found to be 3.72 (2.34–5.90), *P* < 0.00001. The credibility of the study results can be observed in Figure 10.

**Figure 11:**
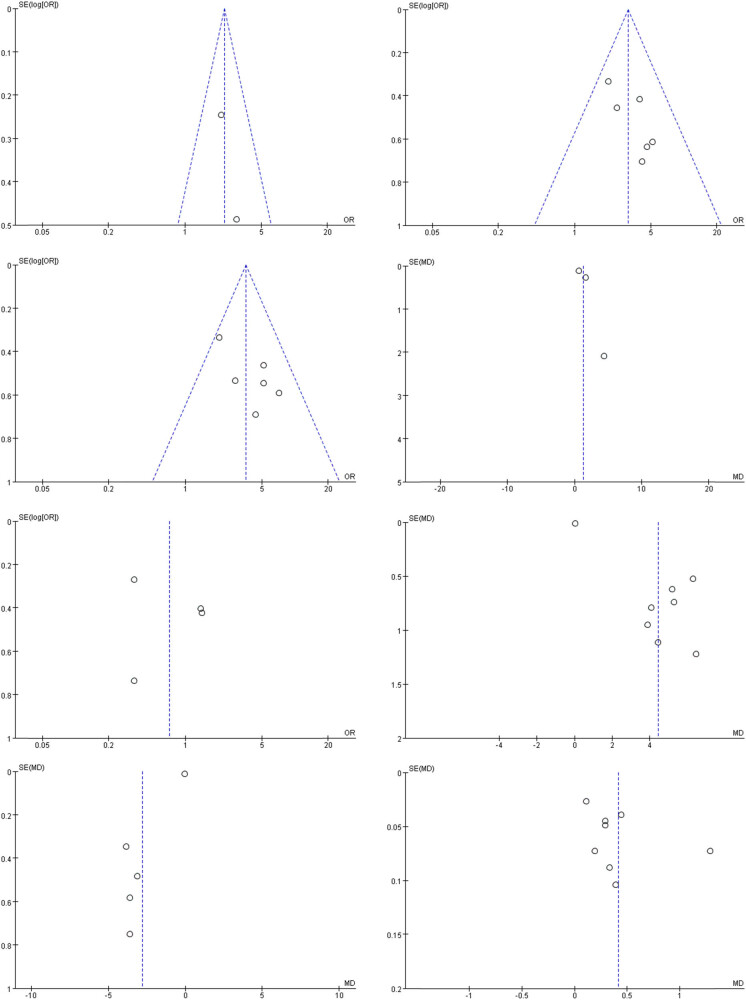
Funnel diagram of test group and control group.

The outcome indicators examined in this study presented inherent biases, and the findings demonstrated an asymmetrical pattern in the funnel plot, suggesting the presence of bias. Refer Figure 11 for visual representation.

## 4 Discussion

Currently, there is a debate surrounding the application of combined immunotherapy and chemotherapy in individuals diagnosed with NSCLC. Therefore, this study aims to quantitatively assess and analyze previous research on the effectiveness and safety of combining immunotherapy and chemotherapy, in order to shed light on this controversial issue.

The results obtained from meta-analysis revealed that the immunotherapy combined with chemotherapy group exhibited significantly higher rates of 1-year OS, ORR, DCR, and PFS compared to the control group. Conversely, there was no notable variation in adverse reactions between the two groups. Concerning immune function, the experimental group demonstrated notably higher levels of CD4+ and CD4+/CD8+ in comparison to the control group. However, the experimental group displayed a lower level of CD8+ compared to the control group. Furthermore, sensitivity analysis demonstrated the stability and reliability of the combined effect (OR (95% CI) 3.72 (2.34–5.90), *P* < 0.00001). The results derived from the funnel chart pointed to the existence of publication bias. In order to reduce the sources of bias analysis, it is recommended to apply more stringent inclusion and exclusion criteria in future studies. In addition, expanding the source of databases, not limited to Chinese and English databases, can also be one of the important ways to reduce bias analysis.

There are several limitations to this study. First, the study includes a small sample size and a limited number of relevant literature sources, which restricts the support from multicenter and large sample studies. Second, individual patients exhibit variations in disease state and progression, leading to potential confounding factors. Lastly, the usage of different immune or chemotherapy regimens and treatment durations may introduce bias into the results of this study.

To summarize, the combination of chemotherapy and immunotherapy exhibits the potential to enhance both survival rates and clinical effectiveness, without the concomitant rise in severe adverse reactions. Additionally, there seems to be a tendency toward enhancing immune function, thereby rendering this approach highly suitable for clinical implementation. Therefore, it is suggested that the combination of chemotherapy and immunotherapy can be used clinically for patients with NSCLC, which can improve the potential of survival and clinical efficacy, and will not be accompanied by an increase in serious adverse reactions. Nevertheless, specific suggestions on the need for more randomized controlled trials with a larger sample size or suggestions for standardizing chemotherapy/immunotherapy combinations in future trials could be added.
